# Fulminant proliferative vitreoretinopathy in syphilitic uveitis

**DOI:** 10.1186/s12348-016-0075-2

**Published:** 2016-02-27

**Authors:** Rafael de Pinho Queiroz, André Vasconcelos Diniz, Daniel Vitor Vasconcelos-Santos

**Affiliations:** Faculdade de Medicina, Universidade Federal de Minas Gerais, Av. Alfredo Balena 190. Sala 199, Belo Horizonte, MG Brazil; Hospital São Geraldo/HC-UFMG, Belo Horizonte, Brazil; Centro Brasileiro de Ciências Visuais, Belo Horizonte, Brazil

**Keywords:** Uveitis, Syphilis, Retinal detachment, Proliferative vitreoretinopathy, Histopathology

## Abstract

**Background:**

Syphilis is a reemerging sexually transmitted disease that can lead to any type of intraocular inflammation. Prognosis of syphilitic uveitis after appropriate therapy is classically regarded as favorable. However, visual threatening complications may develop, rarely including rhegmatogenous/tractional retinal detachment (R/T RD) and proliferative vitreoretinopathy.

**Findings:**

We report 4 patients presenting with complex R/T RD and fulminant proliferative vitreoretinopathy despite treatment among 19 patients with syphilitic posterior uveitis consecutively seen at our uveitis service. Most of these complications occurred during or shortly after antibiotic therapy. All patients presented with significant intraocular inflammation, including vitritis, occlusive retinal vasculitis, and retinal infiltrates (necrotizing retinochoroiditis in six eyes of four patients). Two patients (50 %) tested HIV positive, and the same proportion had inadvertently received high dose oral ± intravenous corticosteroids prior to diagnosis of syphilis. Two patients (three eyes) underwent RD surgical repair. Histopathology of an excised epiretinal membrane disclosed fibroglial tissue, with immature glial cells and metaplastic retinal pigment epithelium, admixed with lymphoplasmacytic infiltrate.

**Conclusions:**

Syphilitic uveitis may be complicated by complex RD/fulminant fibroglial proliferation, occurring during/after treatment. Predisposing factors are currently unknown but may include prior use of corticosteroid, necrotizing retinitis and/or high spirochaetal load. A significant inflammatory component may underlie this fulminant fibroglial proliferation, being possibly amenable to modulation by aggressive anti-inflammatory therapy delivered concurrently with parenteral antibiotics.

## Findings

Syphilis has been reemerging globally, particularly in association with acquired immunodeficiency syndrome (AIDS) and with contemporary changes in sexual practices [1]. The prevalence of syphilitic uveitis parallels this rise on the general incidence of the disease, with novel ocular manifestations, such as inner punctate retinitis, being recently described [2]. The prognosis has been classically regarded as favorable, with good response to parenteral penicillin [1–4].

From April 2011 to October 2012, we had 19 consecutive patients with syphilitic posterior uveitis admitted to our referral hospital, of which 4 (21.05 %) presented with rhegmatogenous/tractional retinal detachment (R/T RD) and fulminant proliferative vitreoretinopathy (PVR) during or shortly after parenteral penicillin therapy.

### Report of cases

#### Case 1

A 24-year-old male reported decreased vision in both eyes, worse in the left (OS), for 8 weeks. Best-corrected visual acuity (BCVA) on admission was 20/63 in the right eye (OD) and hand movements (HM) in OS. Biomicroscopy showed 0.5+ anterior chamber (AC) cells in OD and 2+ AC cells in OS, with 2+ vitreous cells bilaterally. Intraocular pressure (IOP) was within normal limits. Fundus examination revealed 1+ vitreous haze in both eyes (OU). Punctate inner retinal infiltrates (PRI) in the temporal periphery of OD and nasally in OS were also seen, in addition to a large necrotizing lesion involving the inferior aspect of the left retina.

In the face of positive syphilis serology (Table 1), he was admitted and treated with intravenous (IV) penicillin (4 million IU q4h). Oral prednisone 60 mg daily was added 3 days later, in a tapering regimen. Resolution of active intraocular inflammation was verified upon discharge. In the following week, BCVA improved to 20/40 in OD and 20/32 in OS.

**Table 1:** Aspects of four patients with syphilitic uveitis presenting with retinal detachment and fulminant fibroglial proliferation

Case	Initial BCVA	Laboratory results	Presentation of posterior uveitis	Duration of treatment	Outcome after treatment^a^	Final BCVA
Case 1	OD: 20/63	Serum: + VDRL: 1/512 + TPHA	OD: PRI	Intravenous penicillin: 14 days	OD: Macular pucker (21 weeks)	OD: 20/63
	OS: HM	CSF: + VDRL Pleocytosis HIV: negative	OS: PRI, NRC	Prednisone: 60 mg/day (tapering regimen for 7 weeks)	OS: Tractional RD (8 weeks)	OS: CF
Case 2	OD: HM	Serum: + VDRL: 1/1024 + TPHA	OD: NRC	Intravenous penicillin: 21 days	OD: Mixed RD (2 weeks)	OD: LP
	OS: HM	CSF: + VDRL Pleocytosis HIV: positive	OS: NRC	Prednisone: 60 mg/day (tapering regimen for 7 weeks)	OS: Mixed RD (1 week)	OS: 20/150
Case 3	OD: CF	Serum: + VDRL:1/4096 + THPA	OD: NRC	Intravenous penicillin: 21 days	OD: Macular Pucker (5 weeks)	OD: 20/250
	OS: HM	CSF: + VDRL Pleocytosis HIV: negative	OS: PPCR, PRI, NRC	Prednisone: 60 mg/day (tapering regimen for 10 weeks)	OS: Mixed RD (4 weeks)	OS: HM
Case 4	OD: HM	Serum: + VDRL: 1/512 + THPA	OD: NRC	Intravenous penicillin: 21 days	OD: Tractional RD (2 weeks)	OD: NLP
	OS: 20/200	CSF: − VDRL HIV: positive	OS: Optic disc hyperemia, vascular sheathing	Prednisone: 60 mg/day (tapering regimen for 4 weeks)	OS: No lesions	OS: 20/20

*BCVA* best corrected visual acuity, *OD* right eye, *OS* left eye, *HM* hand movements, *VDRL* Venereal Disease Research Laboratory, *TPHA Treponema pallidum* hemagglutination assay, *CSF* cerebrospinal fluid, *HIV* human immunodeficiency virus, *PRI* punctate inner retinal infiltrates, *NRC* necrotizing retinochoroiditis, *RD* retinal detachment CF, counting fingers, *LP* light perception *PPCR*, posterior placoid chorioretinitis, *NPL* no light perception

^a^The time indicated in brackets refers to interval between initial diagnosis and moment that the complication occurred / was detected.

**Fig. 1:**
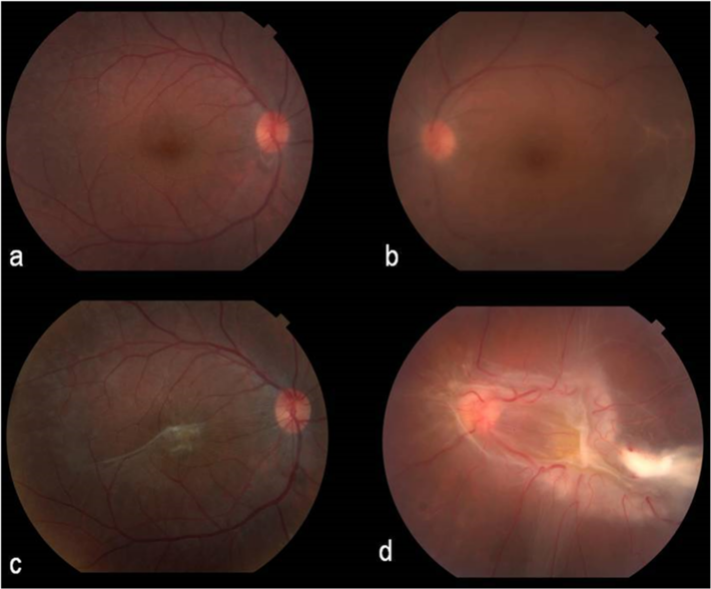
Case 1: Color fundus photographs at baseline (**a** and **b**) and following treatment for neurosyphilis. Macular pucker occurred 19 weeks after discharge in the right eye (**c**) and extensive retinal detachment with fulminant fibroglial proliferation developed 6 weeks after discharge in the left eye (**d**)

Five weeks later, he reported decreased vision in OS since the week prior. BCVA was 20/25 in OD and counting fingers (CF) in OS. Extensive R/T RD in the posterior pole of OS had developed, with subretinal fibrosis and marked epiretinal fibroglial proliferation (Fig 1). The patient refused surgery and RD rapidly progressed over the next few weeks. The contralateral eye retained good vision until 3 months later, when severe macular pucker was noted in OD (Fig. 1), reducing BCVA to 20/63 (Table 1).

#### Case 2

A 45-year-old male was referred with a history of decreased vision in OU for 2 months, despite topical, oral and IV steroids. On admission, BCVA was HM in OU. Biomicroscopy showed large granulomatous keratic precipitates, in addition to 2+ AC cells and 3+ vitreous cells. Fundus examination disclosed 4+ vitreous haze in OU, with dense vitritis and attached retinas on B-scan. Desquamative erythematous lesions of the palms and soles were also observed.

Uveitis workup revealed positive serology for syphilis and for HIV, with CD4+ cell count of 419/mL. The patient was treated for 21 days with IV penicillin, in combination with oral prednisone. Despite improvement of the vitreous haze in OU, R/T RD developed and rapidly progressed, with extensive PVR bilaterally. Pars plana vitrectomy (PPV) was performed in OU, with decreased vision despite attached retinas postoperatively (Table 1).

#### Case 3

A 57-year-old male complained of decreased vision for 30 days in OD, having been unsuccessfully treated for presumed toxoplasmic retinochoroiditis with oral sulfamethoxazole-trimethoprim and prednisone elsewhere. Three weeks later, symptoms started in OS. He also had desquamative erythematous lesions in the palms/soles for several weeks. On admission, BCVA was HM in OD and CF in OS. Biomicroscopy revealed vitreous cells in OU. Fundus examination was precluded because of dense vitreous haze in OD, but B-scan initially ruled out any RD, as well as posterior vitreous detachment (PVD). OS had macular and optic disc edema and PRI in the temporal periphery. Fluorescein angiogram of OS showed changes consistent with superimposed posterior placoid chorioretinitis, as well as multifocal occlusive arteritis.

After positive serology for syphilis (Table 1), the patient received IV penicillin for 21 days. A tapering regimen of prednisone was started 48 h later. Vitreous haze progressively improved thereafter, disclosing foci of retinal necrosis, associated with extensive occlusive vasculitis in OU.

**Fig. 2:**
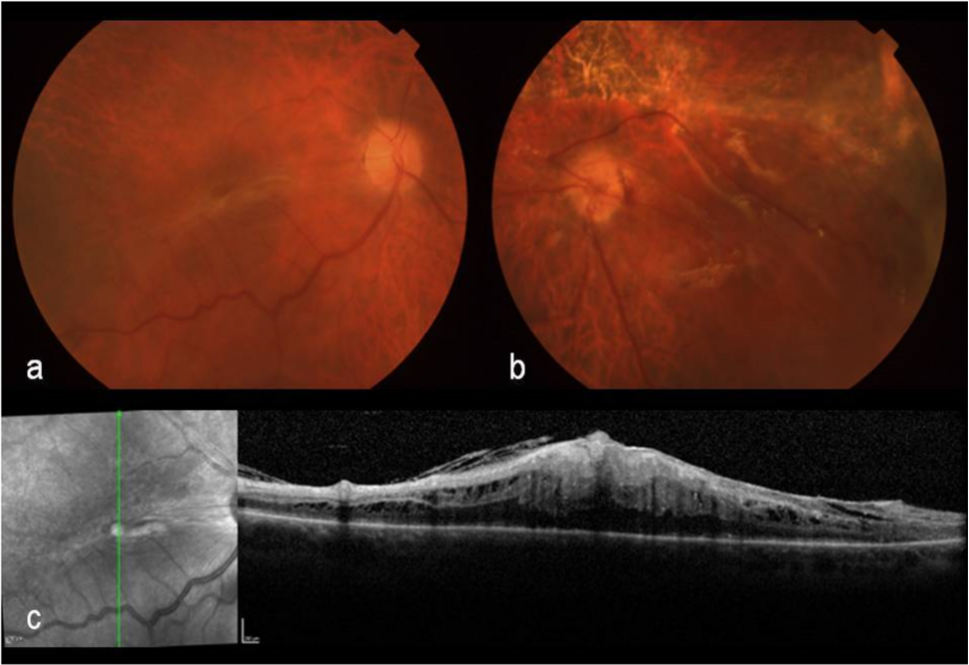
Case 3: Final aspect after treatment. Color fundus photographs (**a**) and optical coherence tomography (**c**) showing macular pucker in the right eye 2 weeks after discharge. Postoperative aspect of mixed retinal detachment in the left eye diagnosed 1 week after discharge (**b**)

**Fig. 3:**
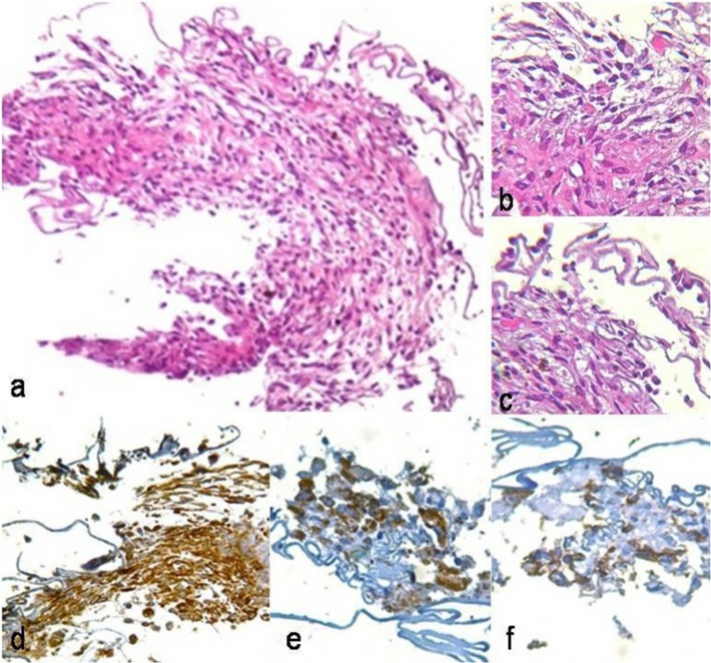
Case 3: Histopathology of excised epiretinal membrane. Hematoxylin and eosin stain shows spindle-shaped (glial) cells as well as lymphoplasmacytic infiltrate (**a**–**c**). Immunohistochemistry shows glial (**d**) staining glial fibrilary acidic protein, retinal pigment epithelial (**e**) staining cytokeratin, and inflammatory (**f**) staining common leukocyte antigen components (original magnification: **a** ×200; **b**–**f** ×400)

Five days after discharge, R/T RD developed in OS. PPV was performed 6 days later. Partial PVD was noted during the procedure, but fulminant PVR had developed (Fig. 2). Histopathology of the excised epiretinal membrane (ERM) disclosed fibroglial proliferation, with immature glial cells and metaplastic retinal pigment epithelium, admixed with lymphoplasmacytic infiltrate. Numerous plasma cells could also be seen adhered to the internal limiting membrane (Fig. 3b, c). In 1 week, marked macular pucker developed in OD, rapidly progressing, and not responding to oral/periocular steroids (Fig. 2a, c).

#### Case 4

A 36-year-old HIV-positive male with CD4+ cell count of 80 cells/mL was referred to our facility complaining of decreased vision OU for 40 days. Biomicroscopy showed 2+ and 1+ AC cells in OD and OS respectively, with 2–3+ vitreous cells OU. Fundus examination of OD revealed 3+ vitreous haze; large necrotizing retinal lesions could also be partially seen inferiorly. OS showed optic disc hyperemia and peripheral vascular sheathing.

Workup revealed positive serology for syphilis (Table 1). IV aqueous penicillin was initiated, with addition of oral prednisone after 48 h. Intraocular inflammation progressively improved, but within 7 days of treatment, R/T RD developed in OD, leading to PVR and progression to no light perception before surgery could be performed. OS maintained good VA (Table 1).

### Comments

Reports since early 2000s have been pointing to the reemergence of syphilis worldwide [1]. Syphilitic uveitis is reported in 2.5–5 % of cases of syphilis [5], manifesting as any type of intraocular inflammation. Despite the usual good prognosis following adequate treatment [1, 4], posterior syphilitic uveitis and panuveitis may potentially be complicated by RD. Eight cases (11 eyes) of patients with syphilitic uveitis presenting with this complication have been previously reported in indexed literature, mostly attributed to a predominant rhegmatogenous component, with variable PVR. Three of these 11 eyes (27.3 %) were left with BCVA ≤20/200 despite surgery [6–9]. We here report even worse outcomes in five eyes of four patients with syphilitic uveitis developing RD/fulminant fibroglial proliferation. Two patients also developed rapidly progressive macular pucker in the contralateral eye (Table 1). Most of these complications occurred during or shortly after antibiotic therapy, except for case 1, in which it surprisingly occurred subacutely, 5 weeks after discharge.

Common features were the presence of necrotizing retinal lesions, high VDRL titers (1:512 to 1:4096; in fact, higher than the majority of other patients) and delayed diagnosis. Nontreponemal antibody titers may indeed correlate with disease activity [10], and this might have been associated with more severe retinal damage in our cases. Characteristic skin lesions in two patients and high VDRL titers in all of them, suggesting high spirochaetal load, point to uveitis occurring at the secondary stage of syphilis. Prior use of corticosteroids and concomitant HIV infection (previously unrecognized in one patient) were probably other contributing factors [3, 11]. It has been demonstrated that corticosteroid-induced immunossupression allows for spirochaetal proliferation [12]. Two of the patients were HIV positive, with a CD4 count of 419 (case 2) and 80 (case 4), and two had received systemic corticosteroids before the diagnosis (Table 1). Another predisposing factor might have been younger age (three of four patients <45 years), possibly associated with incomplete PVD.

Interestingly, histopathology of excised fibroglial membrane disclosed an inflammatory infiltrate rich in plasma cells (Fig. 3), which may underlie the rapid fibroglial proliferation seen in our cases. This pattern is consistent with that seen in the histopathology of Jarisch-Herxheimer reaction following antibiotic therapy [13]. It is likely that such intraocular inflammation in our cases may have served as a trigger for PVR. Indeed, several growth factors (PDGF, FGF, TNF-α, TNF-β) and cytokines (IL-1, IL-6, IL-8, IL-10, INF-γ) are thought to play a role in PVR, mediating chemotaxis and cellular migration, cellular proliferation, membrane formation with remodeling of the extracellular matrix, and, finally, contraction (mediated by myofibroblasts) and subsequent tractional RD [14]. We hypothesize that this deleterious inflammatory response contributing to PVR may be amenable to early modulation by aggressive anti-inflammatory therapy, including higher doses of oral or even periocular costicosteroids.

In conclusion, syphilitic posterior uveitis may be associated with complex RD and fulminant PVR despite therapy, with a potentially poor visual prognosis. Contributing factors, such as delayed diagnosis and previous use of corticosteroids, might be addressed. On the other hand, use of corticosteroids after the initiation of antibiotics might be of benefit to better modulate the inflammatory process, likely associated with a local Jarisch-Herxheimer response.

## Abbreviations

AC: anterior chamber
AIDS: acquired immunodeficiency syndrome
BCVA: best-corrected visual acuity
CF: counting fingers
ERM: epiretinal membrane
HM: hand movements
IOP: intraocular pressure
IV: intravenous
OD: right eye
OS: left eye
OU: both eyes
PPV: pars plana vitrectomy
PRI: punctate inner retinal infiltrates
PVD: posterior vitreous detachment
R/T RD: rhegmatogenous/tractional retinal detachment
